# *Lilium davidii* extract alleviates *p*‑chlorophenylalanine‑induced insomnia in rats through modification of the hypothalamic-related neurotransmitters, melatonin and homeostasis of the hypothalamic-pituitary-adrenal axis

**DOI:** 10.1080/13880209.2020.1812674

**Published:** 2020-09-14

**Authors:** Yanpo Si, Lili Wang, Jinxu Lan, Hanwei Li, Tao Guo, Xiaohui Chen, Chunhong Dong, Zhen Ouyang, Sui-qing Chen

**Affiliations:** a Department of Pharmacy, Henan University of Chinese Medicine, Zhengzhou, China; b Academy of Chinese medicine, Henan University of Chinese Medicine, Zhengzhou, China; c School of Pharmacy, Jiangsu University, Zhenjiang, China

**Keywords:** Hypothalamus, serotonin, γ-aminobutyric acid, melatonin

## Abstract

**Context:**

*Lilium davidii* var. *unicolour *Cotton (*Lilium* genus, Liliaceae) is an edible plant and a herb used in China to alleviate insomnia.

**Objective:**

To investigate the alleviating insomnia mechanism of *L. davidii* (LD).

**Materials and methods:**

Wistar rats were intraperitoneally injected with *p*-chlorophenylalanine (PCPA) to establish an insomnia model. Rats were divided into six groups (*n* = 8): Control, PCPA, Estazolam (0.5 mg/kg), LD extract in low, medium and high doses (185.22, 370.44, 740.88 mg/kg). Serum hormone levels of the HPA axis, levels of 5-HT, NE and MT, and the expression of GABA_A_ and 5-HT1A receptors in hypothalamus were determined. Moreover, behavioural and pathological changes in the hypothalamus were evaluated.

**Results:**

After LD administration, body weight and brain coefficient increased by 2.74% and 8.22%, respectively, and the adrenal coefficient decreased by 25%, compared with PCPA group. Elevation of the serum hypothalamic-pituitary-adrenal (HPA) axis hormone CRH (11.24 ± 3.16 ng/mL), ACTH (565.87 ± 103.44 pg/mL) and CORT (44.28 ± 8.73 ng/mL) in the PCPA group was reversed after LD treatment. Furthermore, abnormal excitatory behaviour [5 min movement distance (2096.34 ± 259.51 cm), central exercise time (5.28 ± 1.08 s)] of insomnia rats in the PCPA group was also relieved. LD extract increased 5-HT and MT levels, reduced NE level in the hypothalamus, and upregulated the expression of GABA_A_ R and 5-HT1A. Moreover, LD extract may improve the pathology of neurons in the hypothalamus.

**Conclusions:**

LD can be considered to develop health-care food or novel drugs to cope with the increasing number of insomniacs.

## Introduction

The importance of a good night’s sleep is well-established all over the world, but the incidence of sleep disorders with insomnia as the main symptom continues to rise with the increase of life pressure, which has become an epidemic in modern society. Insomnia is a widespread malady prevalent in women and the elderly, which can induce extreme medical and psychiatric disorders (Cao et al. 2016). Insomnia is defined by symptoms, including difficulty falling asleep, repeated awakenings with difficulty returning to sleep, or sleep which is non-restorative or poor in quality. It has previously been reported that 27% of people in the world were suffering from insomnia (Doghramji 2006). Sleep disturbances have a significant effect on daily life and reduce the quality of life. Currently, the treatments for insomnia mainly include behavioural therapies and pharmacological treatments. In the USA, the medications approved for treating insomnia represent 4 fundamental pharmacodynamic categories with key actions related to receptors for γ-aminobutyric acid (GABA), melatonin, histamine or orexin/hypocretin. All are based on well-established neurotransmitter effects on sleep and waking (Neubauer et al. 2018). Nevertheless, despite providing effective symptomatic relief, many people suffering from sleep disorders prefer not to use hypnotic drugs (Kim et al. 2019), because the serious side effects may occur with treatment. For example, Benzodiazepine Receptor Agonists (BZRA) enhance the activity of GABA (Rosini and Dogra 2015). As the most widespread inhibitory neurotransmitter in the central nervous system (CNS), GABA has shown targeted actions in hypothalamic regions involved in the regulation of sleep and wakefulness (Saper et al. 2001). The more common side effects associated with BZRA hypnotics include somnolence, dizziness, headache, fatigue, ataxia, anterograde amnesia and confusing behaviours. Furthermore, many plant-based drugs with a relatively low side effect risk have been used in the treatment of insomnia (Wheatley 2005). Therefore, it is warranted to develop healthy food and new bioactive substances derived from natural sources that present with similar efficacy but fewer side effects compared to hypnotic drugs.

Serotonin (5-HT), found in mammalian tissues, especially in the cerebral cortex and synapses (known as serotonin), is an inhibitory neurotransmitter that can bring mental pleasure (Shi et al. 2018). Melatonin (MT), also known as a pineal voxel, is a steroid hormone that is secreted by the pineal gland, and has calming hypnosis effects and regulates the sleep awakening cycle.

*Lilium davidii* var. *unicolour* Cotton (Liliaceae) has high edible value and is a variant of Chuan lily, mainly cultivated in Lanzhou city of the Gansu province. It is known for its large size, white and thick flesh, and sweetness. *L. davidii* (LD) is ‘the only sweet lily’ in the country of China and Southeast Asia (Zhang et al. 2010). Its bulbs, in China referred to as ‘Baihe’, are regularly consumed as food for their distinctive taste and have been used as a herb in Chinese folk medicine as a remedy for insomnia and dreamful sleep. Some soups prepared with Baihe as the main ingredient, including Baihe Lianzi soup and Gouqi Baihe soup, are used to relieve insomnia in Chinese people. The bulbs of LD are rich in phospholipids, sugar, amino acids, crude fibres, vitamins, pantothenic acid, carotene and other nutrients, as well as various bioactive metabolites, such as polysaccharides, saponins, flavonoids and alkaloids (Wang 2007).

Intraperitoneal injection of *p*-chlorophenylalanine (PCPA) induced sleep deprivation in rodents is the most commonly used model to study the underlying in the mechanism of insomnia. PCPA, a 5-HT synthesis inhibitor, can selectively act on tryptophan hydroxylase by inhibiting enzyme activity and hindering 5-HT synthesis, resulting in the disappearance of sleep circadian rhythm, almost complete insomnia, while 5-HT content is reduced, accompanied by hypothalamic-pituitary-adrenal axis (HPA) dysfunction (Quan et al. 2006; Shi et al. 2018). It was reported that *Lilium brownii* F. E. Brown var. *viridulum* Baker and *Lilium lancifolium* Thunb can significantly enhance GABA-induced chloride currents (I_GABA_) (Singhuber et al. 2012). At present, no reports are available on the underlying mechanism of alleviating insomnia for LD. Therefore, the aim of this study was to investigate the effects of LD on PCPA-induced insomnia rats, and to explore the underlying mechanism of alleviating insomnia.

## Materials and methods

### Plant material and plant extract preparation

Fresh lily bulbs were purchased from the Qilihe district of Lanzhou city, Gansu province in April 2019, and were identified as bulbs of *Lilium davidii* var.* unicolour *Cotton by Prof. Ai-Hong Zhao (Lanzhou University of Technology). A voucher specimen 20190428 A was deposited at the Henan University of Chinese Medicine (Henan, China). Fresh lily bulbs were peeled and dried for 36 h at 60 °C for experimental use. Processed dry lily bulbs were stored in a dry environment before measurements. Preliminary experiments showed that 60% acetone extract could better prolong the sleep time of insomnia rats. Dried plant material was extracted three times (material to liquid ratio 1:8; for 14 d, 14 d and 21 d) by maceration in 60% acetone at room temperature and concentrated *in vacuo* using a rotary evaporator, after which a brown extract was obtained. The yield was 13.23% (w/w).

### Experimental animals

Prior authorization for the use of laboratory animals in this study was obtained from the Regulations of Experimental Animal Administration issued by the State Committee of Science and Technology of the People’s Republic of China. The animal experiment research content of the study was supervised by the Experimental Animal Ethics Committee of the Henan University of Chinese Medicine (Henan, China), which was in line with animal protection, animal welfare, ethical principles and the relevant provisions of the National Laboratory Animal Welfare Ethics. Experiments were implemented using specific pathogen-free (SPF) male Wistar rats (160 − 180 g), purchased from Beijing VitalRiver Laboratory Animal Technology Co., Ltd. [license number: SCXK (Beijing, China) 2012 − 0006]. Animals were kept under standard room conditions (temperature 25 ± 2 °C and relative humidity 50 ± 5% with 12 h light/dark cycles) with standard rodent diet and water *ad libitum*. Animals were deprived of food 12 h before experiments.

The insomnia animal model was established according to a previously reported method (Yan et al. 2019). After acclimation period of a week, 48 male Wistar rats were divided into six groups (*n* = 8 per group) as follows: Control group (untreated, only received saline), PCPA group (400 mg/kg), PCPA + Estazolam group (0.5 mg/kg), PCPA + LD extract [low (149.66 mg/kg), medium (299.32 mg/kg) and high (598.64 mg/kg) doses]. PCPA (Sigma, St. Louis, MO, USA; No: C6506) was suspended in weakly alkaline saline (PH 7 ∼ 8) and administered intraperitoneally (i.p. 400 mg/kg) once a day for 2 days, except the Control group, which was given the same amount of normal saline. After i.p. injection of 28 − 30 h, the circadian rhythm of rats disappeared. The overall observation was significantly different from the Control group, indicating that the model was successfully replicated. On the third day, rats in PCPA + LD extract group and PCPA + Estazolam group were given different doses of LD extract and 0.5 mg/kg Estazolam by oral gavage once a day for 7 days, respectively. Rats in the other two groups were administered the same volume of normal saline by oral gavage. The open-field test was conducted to determine the exploring ability in new environments.

### Bodyweight and organ coefficient

Rats were weighed before each administration for 10 consecutive days. Rats were anaesthetized by i.p. injection of 10% chloral hydrate and their adrenals were excised after blood was taken through the abdominal aorta. The whole brain was quickly removed on ice and was weighed to calculate the brain coefficient. The organ coefficient of animals is the ratio of organ weight to body weight. The changes in organ coefficients often reflect the comprehensive toxicity of chemical drugs to an organ, which can be evidence of the possibility of histopathological changes (Huang et al. 2003).

### Open field test

During the open field test, rats were placed in the centre of a metal box of the open field experimental device. After recording the number, date and status of the rats in the operating software, the recording system with a nine-square grid mode and central area ratio of 0.3 was turned on, and the video tracking analysis system was used to automatically record the activity of each rat during 5 min for calculating the exercise distance and exercise time. Each rat was tested one time during the experiment (Liang 2019). After each animal was tested once, the recorder was cleaned before measurements were performed on the next rat.

### Assay for serum levels of CRH, ACTH and CORT

Rats were anaesthetized by i.p. injection of 10% chloral hydrate, and rat blood samples obtained through the abdominal aorta (8 rats per group) were centrifuged at 3500 rpm at 4 °C for 15 min. Next, the levels of corticotropin-releasing hormone (CRH), adrenocorticotropic hormone (ACTH) and corticosterone (CORT) in serum were determined using the commercial kits (Elabscience Biotechnology Co., Ltd. Wuhan, China) according to the manufacturer’s protocols.

### Assay for hypothalamic 5-HT, NE and MT

Rats were anaesthetized by i.p. injection of 10% chloral hydrate and blood was taken through the abdominal aorta. The whole brain was quickly removed on ice after the head of each rat was cut off by the guillotine method, and then the hypothalamus was separated by precision forceps. The supernatant of the hypothalamus was homogenized and centrifuged at 5000 *g* for 10 min to determine the levels of 5-HT, Norepinephrine (NE), and MT by ELISA kits (Elabscience Biotechnology Co., Ltd. Wuhan, China).

### Histological observation

PCPA-induced hypothalamic lesions were evaluated using haematoxylin and eosin (H&E) staining. In brief, the hypothalamus of each rat was fixed in 10% formalin solution, dehydrated with a series of ethanol solutions from 50% to 100%, cleared in xylene, and embedded in paraffin. Hypothalamic tissue was sliced into 5 μm sections, then stained with H&E and pathological changes were evaluated using a light microscope at a magnification of 200×.

### Western blot analysis

Whole hypothalamic tissue protein lysates were prepared in radioimmunoprecipitation assay (RIPA) lysis buffer (Servicebio Biotechnology, Wuhan, China) in the presence of protease inhibitors. Protein samples (40 μg per sample) were loaded on 10% SDS-polyacrylamide gels to separate the target proteins and transferred to polyvinylidene fluoride (PVDF) membranes (Millipore, Bedford, MA, USA). Subsequently, membranes were washed with Tris-buffered saline containing 0.1% Tween-20 (TBST), blocked with 5% non-fat dry milk in TBST for 1 h at 37 °C, and probed with primary antibodies. Followed by three washes with TBST after incubation at 4 °C overnight, the membranes were incubated with horseradish peroxidase (HRP)-conjugated antibodies for 1 h at room temperature. Protein bands were visualized using an electrochemiluminescence (ECL) system. Finally, the densities of the bands were determined using the Alpha Innotech alphaEaseFC luminescent image analyser (Alpha Innotech, Santa Clara, CA, USA), and the optical density of the protein bands was quantified by Image J software and normalized to GAPDH (glyceraldehyde-3-phosphate dehydrogenase).

### Statistical analysis

The results of animal experiments were expressed as means ± standard error ($\bar{X}\pm S$). One-Way ANOVA (Analysis of Variance) was used to compare the results of multiple groups. *p* < 0.05 indicates a significant difference, and *p* < 0.01 indicates a very significant difference. All data were processed using Statistical Product and Service Solutions (SPSS) 18.0 statistical software.

## Results

### Body weight and brain, and adrenal coefficient

**Figure 1.**
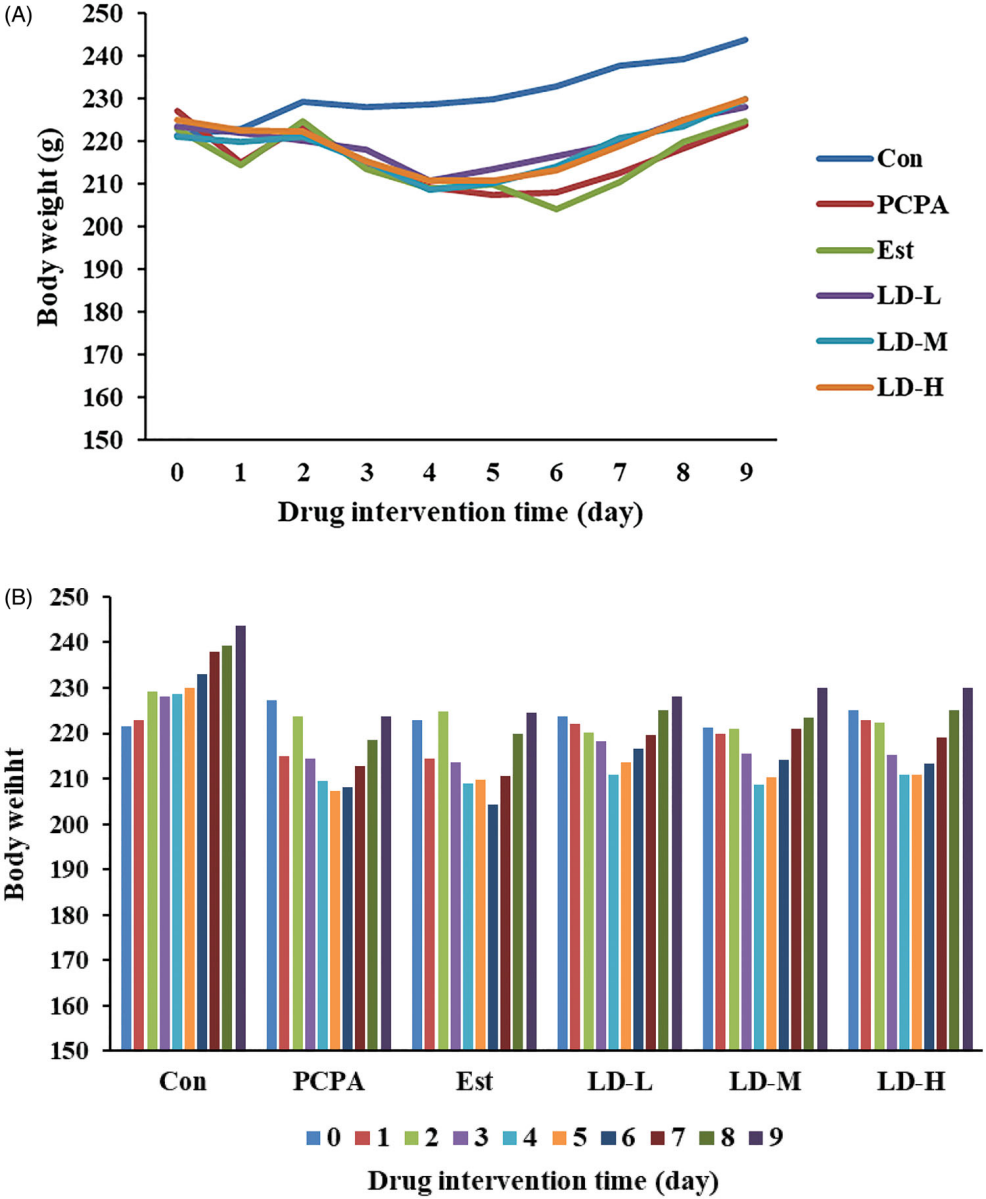
Effect of LD extract on body weights (A, line graph; B, Bar graph) in PCPA-induced insomnia rat. The body weight of the animals is weighed for 10 consecutive days. Animals were intraperitoneally injected PCPA for the first two days and administered by gavage for 7 days.

The results showed that there was a difference in body weight gain and the adrenal coefficient between the Control and PCPA group (Figure 1, Table 1). When compared to the PCPA group (0.73 ± 0.04), a significant increase was observed in body weight and brain coefficient (LD-M, 0.79 ± 0.03 and LD-H, 0.79 ± 0.02), as well as a decrease in adrenal coefficient (LD-M, 0.012 ± 0.002 and LD-H, 0.013 ± 0.002) in rats treated with LD extract.

**Table 1.** Effects of LD extract on brain coefficient and adrenal coefficient in PCPA-induced insomnia rat ($\bar{\text{x}}$ ± s, *n* = 8).

Group	Dosage(mg/kg)	Brain coefficient	Adrenal coefficient
Con	−	0.75 ± 0.03	0.010 ± 0.004
PCPA	400	0.73 ± 0.04	0.016 ± 0.002**
Est	0.5	0.80 ± 0.04**^#^**	0.014 ± 0.002
LD-L	185.22	0.77 ± 0.04	0.014 ± 0.001
LD-M	370.44	0.79 ± 0.03**^#^**	0.012 ± 0.002**^##^**
LD-H	740.88	0.79 ± 0.02**^#^**	0.013 ± 0.002**^#^**

Results expressed as the means ± SE (*n* = 8). Statistical differences between groups were analysed statistically by One-Way ANOVA. *p < 0.05 and **p < 0.01 compared with Con, **^#^***p* < 0.05 and **^##^***p* < 0.01 compared with PCPA.

### Effects of LD extract administration on the open field test

**Figure 2.**
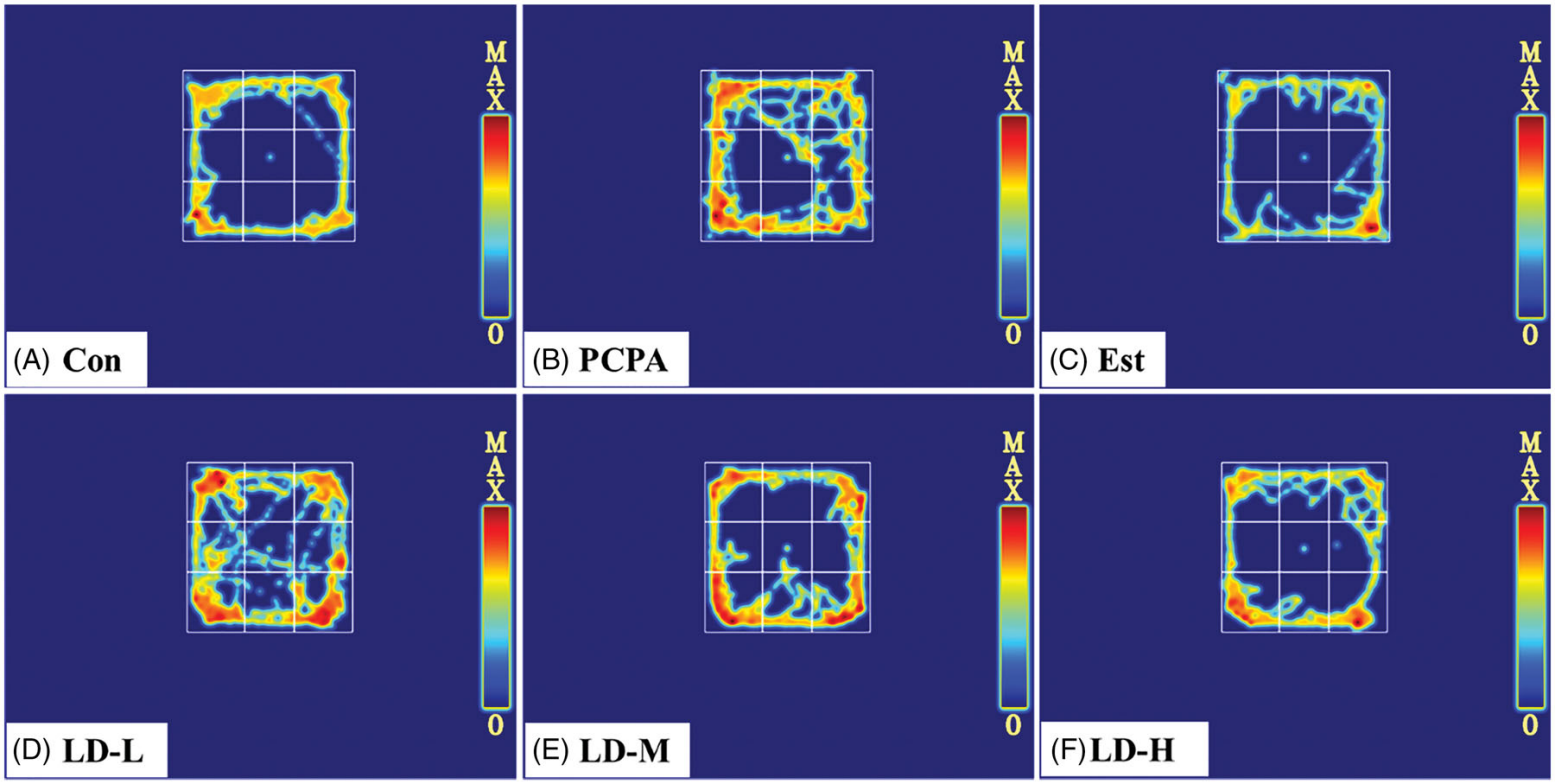
The trajectories in the heat map represents the movement distance of the mouse within 5 minutes, and the movement distance and exercise time within 5 minutes are quantified by the video tracking analysis system.

The effect of LD extract on the behaviour of insomnia rats was measured by the open field test. As shown in Table 2 and Figure 2, the LD extract at the middle (LD-M, 370.44 mg/kg) and high dose (LD-H, 740.88 mg/kg) significantly reduced the 5 min movement distance (LD-L, 1610.43 ± 457.49 cm; LD-M, 1446.18 ± 373.43 cm; and LD-H, 1625.86 ± 386.12 cm) and central exercise time (*p* < 0.05 or 0.01) of rats. Taken together, the results of the behavioural study revealed that LD extract reduced the abnormal excitement induced by PCPA injection.

**Table 2.** Effect of LD extract on behavioural in PCPA-induced insomnia rat ($\bar{\text{x}}$ ± s, *n* = 8).

Group	Dosage (mg/kg)	Movement distance (cm)	Central exercise time (s)
Con	−	1583.34 ± 236.1	3.70 ± 0.88
PCPA	400	2096.34 ± 259.51**	5.28 ± 1.08**
Est	0.5	1569.24 ± 338.86^##^	3.37 ± 0.54**^##^**
LD-L	185.22	1610.43 ± 457.49**^#^**	3.52 ± 0.74**^##^**
LD-M	370.44	1446.18 ± 373.43**^##^**	3.59 ± 0.78**^##^**
LD-H	740.88	1625.86 ± 386.12**^#^**	3.71 ± 0.83**^##^**

Data were expressed as means ± SE (*n* = 8). *p < 0.05, **p < 0.01 compared with the Con. ^#^*p* < 0.05, ^##^ *p* < 0.01 compared with the PCPA group.

### Effects of LD extract on serum levels of CRH, ACTH and CORT in PCPA-induced insomnia rats

**Figure 3.**
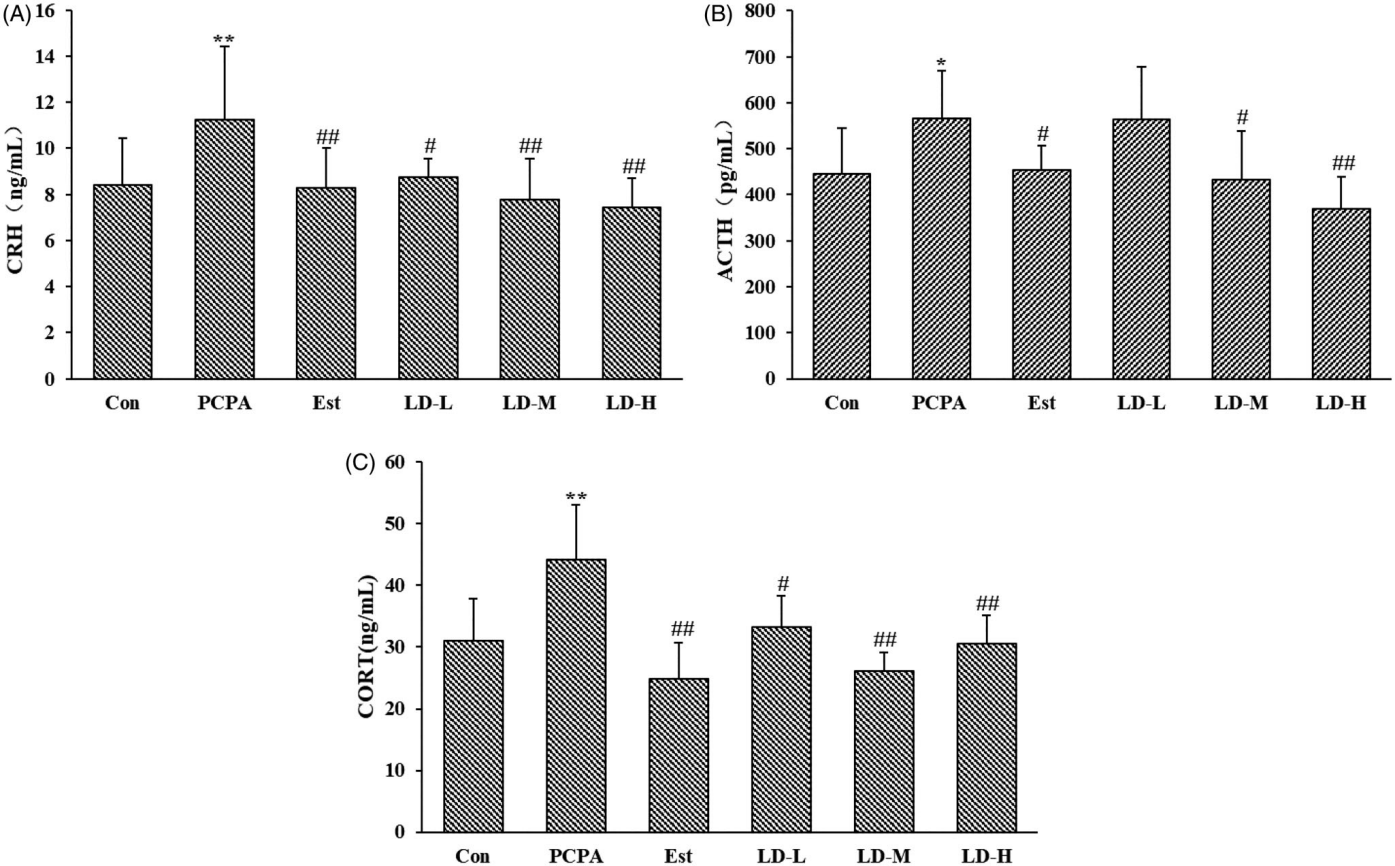
Effects of LD extract on serum levels of CRH (A), ACTH (B) and CORT (C) in PCPA-induced insomnia rat. Control: received only saline treatment; PCPA: treated with 400 mg/kg PCPA only; Est: PCPA + 0.5 mg/kg Estazolam; LD-L: PCPA + 185.22 mg/kg *L. davidii* extract; LD-M: PCPA + 370.44 mg/kg *L. davidii* extract; LD-H: PCPA + 740.88 mg/kg *L. davidii* extract. Values are presented as the means ± standard error (SE) for each group, *n* = 8. Different letters indicate significant differences (*p* < 0.05 or 0.01) among samples, *p < 0.05, **p < 0.01 compared with Control; ^#^*p* < 0.05, ^##^*p* < 0.01 compared with PCPA using One-Way ANOVA.

As shown in Figure 3(A–C), compared with the Control group, PCPA administration significantly increased serum levels of CRH (11.24 ± 3.16 ng/mL), ACTH (565.87 ± 103.44 pg/mL) and CORT (44.28 ± 8.73 ng/mL) (*p* < 0.05 or 0.01). In addition, oral administration of LD extract over 7 days produced a significant (*p* < 0.05 or 0.01) decrease in CRH (LD-L, 8.76 ± 0.78 ng/mL; LD-M, 7.78 ± 1.78 ng/mL; LD-H, 7.45 ± 1.25 ng/mL) and CORT (LD-L, 33.3 ± 5.09 ng/mL; LD-M, 26.13 ± 2.99 ng/mL; LD-H, 30.50 ± 4.71 ng/mL) levels at all tested doses, and a significant decrease in the ACTH level at high (433.02 ± 105.89 pg/mL) and middle (369.37 ± 69.34 pg/mL) doses. These beneficial effects were comparable with that of 0.5 mg/kg Estazolam, which is a benzodiazepine drug that binds the benzodiazepine receptor and enhances GABA effects.

As shown in Figure 3(A–C), compared with the Control group, PCPA administration significantly increased serum levels of CRH (11.24 ± 3.16 ng/mL), ACTH (565.87 ± 103.44 pg/mL) and CORT (44.28 ± 8.73 ng/mL) (*p* < 0.05 or 0.01). In addition, oral administration of LD extract over 7 days produced a significant (*p* < 0.05 or 0.01) decrease in CRH (LD-L, 8.76 ± 0.78 ng/mL; LD-M, 7.78 ± 1.78 ng/mL; LD-H, 7.45 ± 1.25 ng/mL) and CORT (LD-L, 33.3 ± 5.09 ng/mL; LD-M, 26.13 ± 2.99 ng/mL; LD-H, 30.50 ± 4.71 ng/mL) levels at all tested doses, and a significant decrease in the ACTH level at high (433.02 ± 105.89 pg/mL) and middle (369.37 ± 69.34 pg/mL) doses. These beneficial effects were comparable with that of 0.5 mg/kg Estazolam, which is a benzodiazepine drug that binds the benzodiazepine receptor and enhances GABA effects.

### Effects of LD extract on hypothalamic 5-HT, NE and MT in PCPA-induced insomnia rat

**Figure 4.**
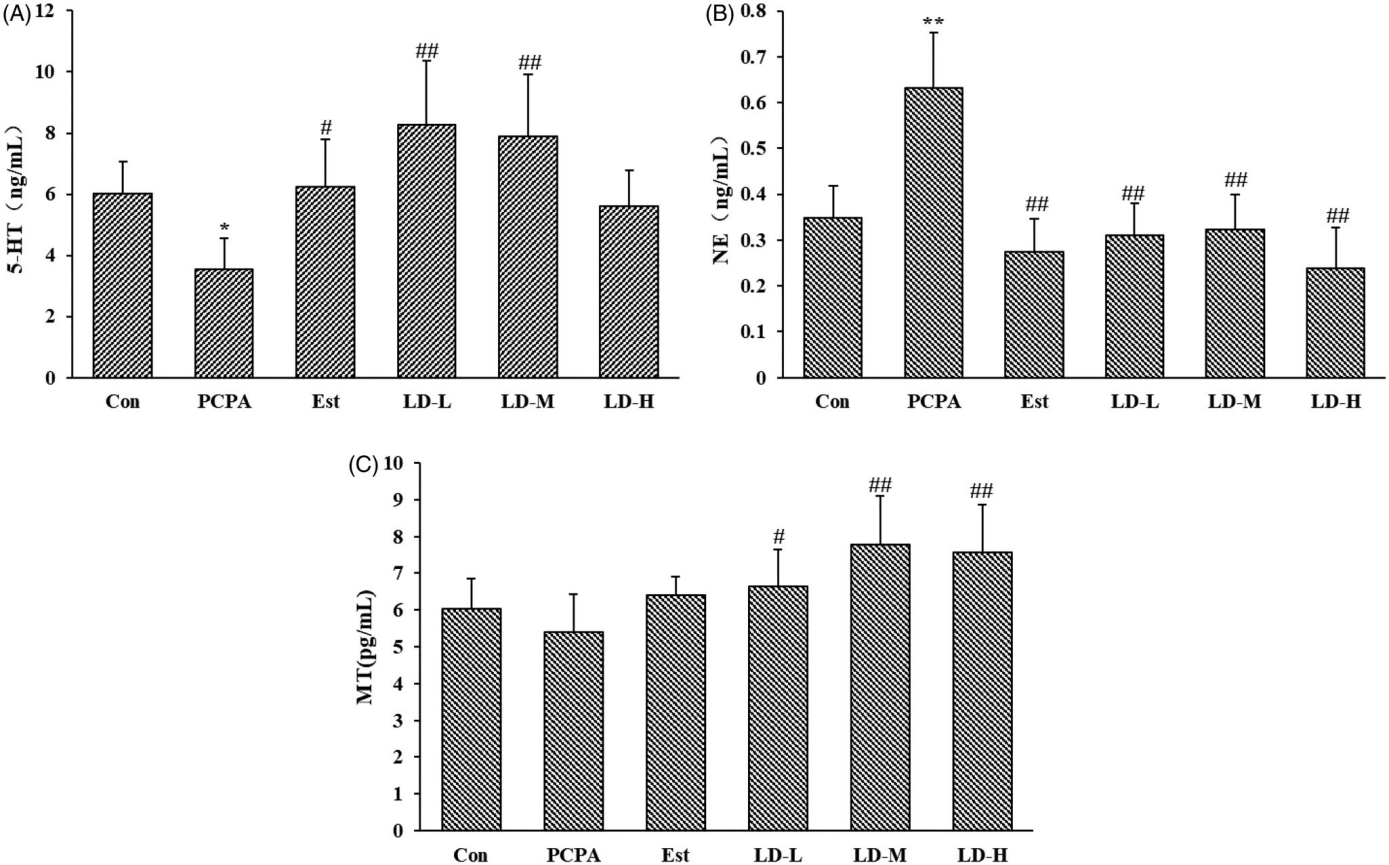
Effects of LD extract on hypothalamic levels of 5-HT (A), NE (B) and MT (C) in PCPA-induced insomnia rat. Control: received only saline treatment; PCPA: treated with 400 mg/kg PCPA only; Est: PCPA + 0.5 mg/kg Estazolam; LD-L: PCPA + 185.22 mg/kg *L. davidii* extract; LD-M: PCPA + 370.44 mg/kg *L. davidii* extract; LD-H: PCPA + 740.88 mg/kg *L. davidii* extract. Values are presented as the means ± standard error (SE) for each group, *n* = 8. Different letters indicate significant differences (*p* < 0.05 or 0.01) among samples, *p < 0.05, **p < 0.01 compared with Control; ^#^*p* < 0.05, ^##^*p* < 0.01 compared with PCPA using One-Way ANOVA.

As shown in Figure 4(A–C), PCPA administration significantly decreased the level of 5-HT (3.58 ± 1.00 ng/mL, *p* < 0.05) and increased the level of NE (0.63 ± 0.12 ng/mL, *p* < 0.01) in the hypothalamus, while no difference in the MT level (5.41 ± 1.03 pg/mL) was observed when compared with the Control group. In addition, oral administration of LD extract at all tested doses for 7 days produced a significant (*p* < 0.01) increase in the MT level (LD-L, 6.63 ± 1.03 pg/mL; LD-M, 7.77 ± 1.32 pg/mL; LD-H, 7.57 ± 1.28 pg/mL) and a decrease in NE (LD-L, 0.31 ± 0.070 ng/mL; LD-M, 0.32 ± 0.077 ng/mL; LD-H, 0.24 ± 0.088 ng/mL), at the middle (LD-M, 7.88 ± 2.02 ng/mL) and low doses (LD-L, 8.27 ± 2.08 ng/mL), and increase only in the 5-HT level.

### Histopathological observation

**Figure 5.**
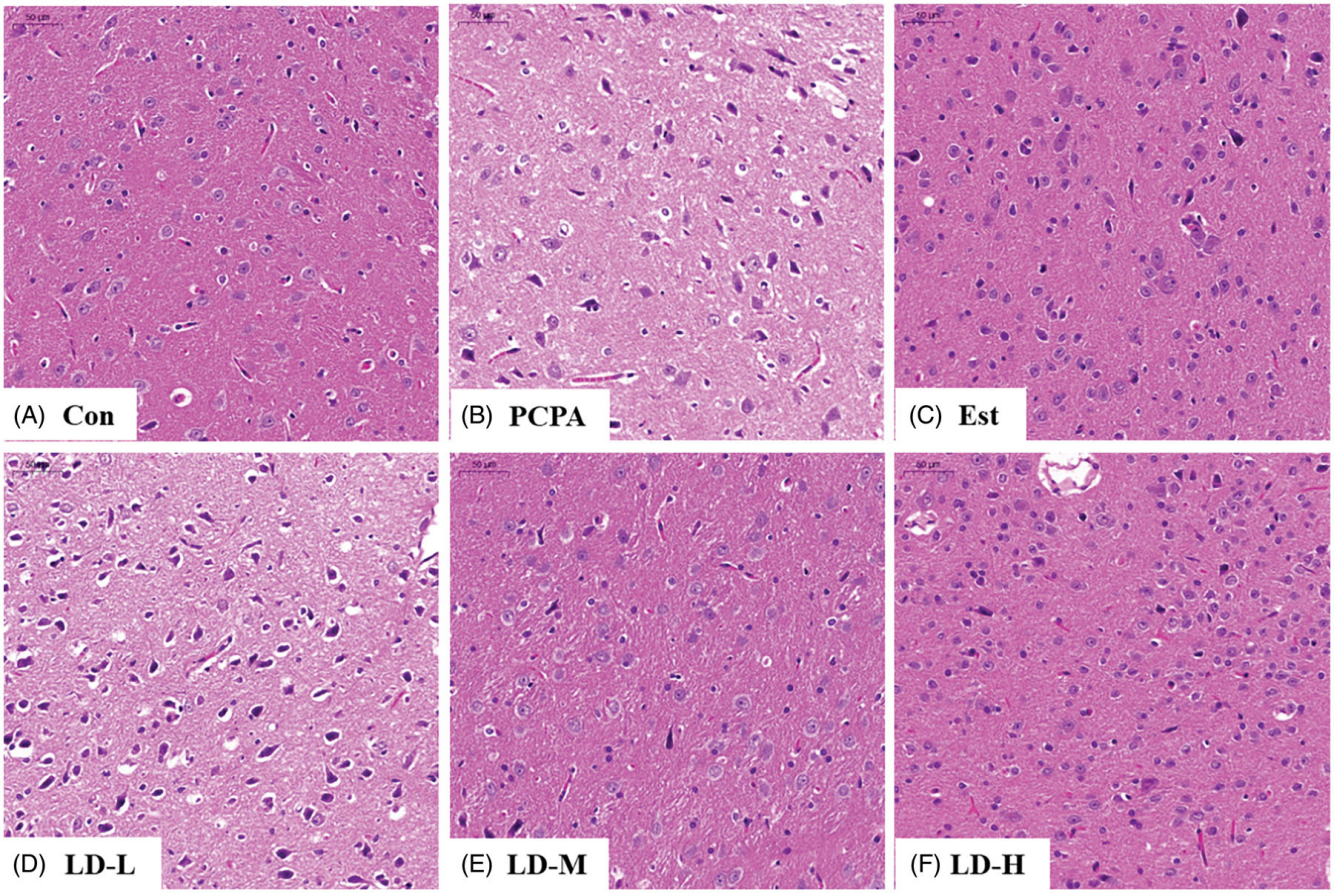
Histopathological observation of hypothalamic slices in rat of PCPA-induced insomnia. Hypothalamic sections were stained with haematoxylin and eosin (H&E stain 200 ×). (A) Con: Control (untreated); (B) PCPA (400 mg/kg alone); (C) Est: PCPA + 0.5 mg/kg Estazolam; (D) LD-L: PCPA + 185.22 mg/kg *L. davidii* extract; (E) LD-M: PCPA + 370.44 mg/kg *L. davidii* extract; (F) LD-H: PCPA + 740.88 mg/kg *L. davidii* extract. *p < 0.05, **p < 0.01 compared with Control; ^#^*p* < 0.05, ^##^*p* < 0.01 compared with PCPA using One-Way ANOVA.

As shown in Figure 5(A–F), hypothalamic nerve cells in the Control group were abundant, and the various cell structures were intact, evenly distributed and visible. In contrast, hypothalamic nerve cells in the PCPA group were loosely arranged, severely deformed, and there was a large number of debris cells. Cells in the LD administration group were neatly arranged, and a large number of atrophic cells were recovered. The pathological changes were also consistent with the data shown in literature reports (Liu and Wang 2015). The recovery of hypothalamic cells in the LD-M and LD-H groups was more significant.

### Effects of LD extract on hypothalamic GABA_A_ R and 5-HT1A in PCPA-induced insomnia rat

**Figure 6.**
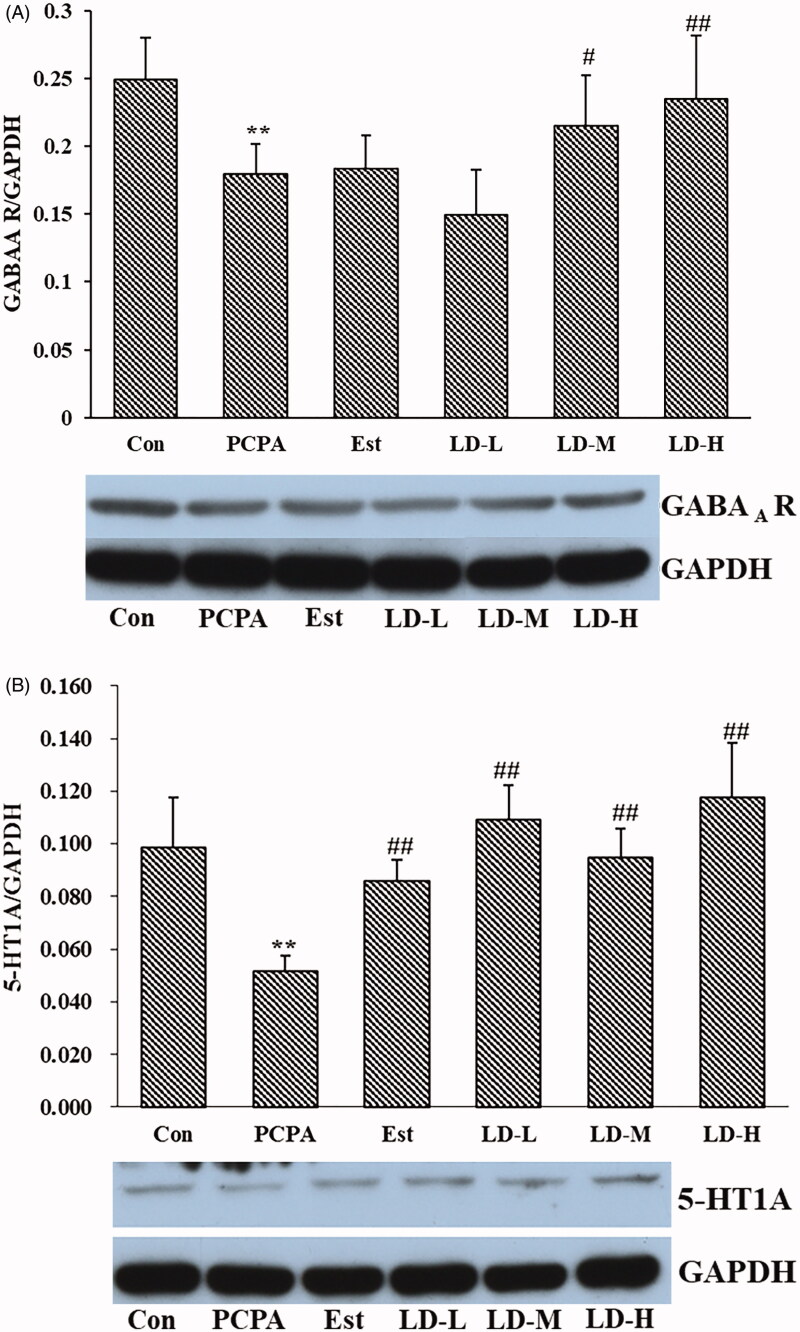
Effect of LD extract on the expressions of GABA_A_ R (A), 5-HT1A (B) in PCPA-stimulated insomnia rat. GABA_A_ R and 5-HT1A protein expression were determined by Western blotting. The data presented are means ± SE of three independent experiments. *p < 0.05, **p < 0.01 compared with Control; ^#^*p* < 0.05, ^##^*p* < 0.01 compared with PCPA using One-Way ANOVA.

As shown in Figure 6(A,B), PCPA administration significantly decreased the level of 5-HT1A and GABA_A_ R in the hypothalamus (*p* < 0.01). Our data showed that oral administration of LD extract (370.44 mg/kg) produced a significant (*p* < 0.01) increase in the levels of 5-HT1A and GABA_A_ R.

## Discussion

Sleep is considered an important process because of its unique role in maintaining and performing normal physiological activities. Sleep is regulated by the circadian rhythm and homeostatic mechanisms, which are dominated by the CNS (Shi et al. 2014; Zhang et al. 2018). In the brain, there are many endogenous neurotransmitters, including monoamines, noradrenergic and cholinergic neurotransmitters that are involved in sleep mechanisms (Zeng et al. 2018). To investigate the activity and characteristics of LD extract on alleviating insomnia, the PCPA-induced insomnia test and spontaneous exploration activities were performed. The PCPA-induced insomnia rat model is the most commonly used classic model to study the mechanism of insomnia, which can deplete the 5-HT content in the brain, and is accompanied by dysfunction of the HPA axis (Hu et al. 2016).

As shown in Figure 1, the weights of rats in the Control group gradually increased with time, while in the PCPA group, body weight decreased significantly within a few days. Moreover, we found that the food intake of each rat was significantly reduced within a few days after the i.p. injection of PCPA. Using the 16S RNA sequencing method, we demonstrated that the i.p. injection of PCPA changed the composition and ratio of the intestine flora, which was accompanied by a decrease in food intake and weight loss from the PCPA group. It is hypothesized that these changes might be significantly recovered through LD extract intervention (relevant results have not yet been published). Indicating the effect of alleviating insomnia of *L. davidii* may be related to the regulation intestinal flora. Moreover, the brain coefficient of rats in the PCPA group decreased to a certain extent (however no significant difference), and the adrenal coefficient increased significantly (*p* < 0.01). The reduction in rat brain coefficients may to a certain extent be characterized by loosely arranged neurons, blurred cell boundaries and a large number of atrophic neurons in the hypothalamus after the i.p. injection of PCPA (Figure 5). The abnormal increase of adrenal glands may also be related to the dysfunction of the HPA axis. Obviously, in the LD extract group, rats gradually gained weight (Figure 1), their brain coefficient significantly increased (*p* < 0.05), and the adrenal coefficient reduced (*p* < 0.01) (Table 1). The pathology of the hypothalamus in the LD extract group also was significantly improved (Figure 5).

The spontaneous exploration activities of rats can indirectly reflect the inhibitory effect of drugs on the CNS (Barros et al. 1991), which is the preliminary pharmacodynamic evaluation method of sedative-hypnotic drugs. In this study, the open-field test was used to explore animals in a new environment. Our data demonstrated that the rats showed abnormal excitement after i.p. injection of PCPA (Figure 2 and Table 2) because the 5 min movement distance and the central exercise time in the open field test were significantly increased (*p* < 0.01). This was also a typical feature of PCPA-induced insomnia in rats. Thus, LD extract significantly improved abnormal behaviours.

The HPA axis is an important physiological basis to maintain homeostasis and regulate stress responses. Numerous experiments studies have shown that insomnia animals present with over-activation of the HPA axis. The HPA axis, which starts from the hypothalamus and stimulates the production of glucocorticoids, is the main stress axis of the body. The dysfunction of the HPA axis is mainly caused by increased expression of CRH, ACTH and CORT (Sun et al. 2020). Activation of the HPA axis exhibits a significant circadian rhythmicity, which is roughly parallel to the activity cycle. CRH secreted by the hypothalamus is a critical factor for regulating the stress response, which directly acts on the pituitary to promote the secretion of ACTH to release glucocorticoids. Glucocorticoids, the primary end products of the HPA axis, represent classic examples of allostatic mediators whose effects involve modulation of the HPA axis to return the system to homeostasis. ACTH promotes adrenal cortex production and secretion of cortisol, while cortisol in turn affects the pituitary and hypothalamus, and weakens hypothalamic secretion activity when the secretion level of corticosteroids is too high (Lo Martire et al. 2019). Therefore, maintaining relative balance and homeostasis of the HPA axis for regulating sleep and the rhythm of awakening is of great significance under the negative feedback regulation mechanism of corticosteroids. HPA axis hormone has clinically been used as an important marker for the diagnosis and treatment of insomnia and other mental disorders (Zhao et al. 2018). In this study, serum levels of HPA axis hormones (CRH, ACTH, CORT) in the PCPA group were higher when compared to that in the normal group (Figure 3). Furthermore, as mentioned before, the adrenal coefficient in the PCPA group was also significantly elevated, and the abnormally increased adrenal gland weight may be related to an increased serum level of CORT, which was secreted by the cortical part of the adrenal gland. Both excessive activation of the HPA axis and the adrenal coefficient of insomnia rats were significantly decreased after 7 days of LD extract intervention (Figure 3 and Table 1). These results suggested that LD extract could play a role in the treatment of insomnia by regulating the function of the HPA axis.

The disturbance of neurotransmitters in the brain is widely approbated to be associated with insomnia in the complex pathogenesis of insomnia (Chokroverty 2017). Various neurotransmitters including NE, dopamine (DA), GABA and 5-HT play important roles in maintaining wakefulness and sleepiness (Yan et al. 2019). Sleep regulation is primarily focussed on monoamine neurotransmitters, mainly include DA, NE, 5-HT, 5-hydroxy indole acetic acid (5-HIAA), etc., which can participate in various physiological activities in our body (Nishino 2013). The diverse effects of 5-HT in sleep regulation are in part because 5-HT can act in different areas of the brain that have been associated with the control of sleep and wake (Zhao et al. 2004). In mammals, sleep states are associated with decreased activity or inactivity of serotonergic neurons (compared to wakefulness). The main functions of 5-HT are to promote wakefulness and to inhibit Rapid Eye Movement Sleep (Dos Santos et al. 2015). NE as a neurotransmitter belonging to the class of catecholamines is mainly synthesized and secreted by sympathetic postganglionic neurons and brain adrenergic nerve endings, and is the main transmitter released by the latter. The level of NE is closely related to the normal operation of the sleep system. In this study, the level of 5-HT observed in the PCPA group was significantly lower when compared to that in the Control group, whereas the MT level was reduced but not significantly different. In addition, the NE level was increased significantly. Levels of 5-HT, MT and NE were reversed by treatment with LD extract (Figure 4), indicating that LD extract may affect serotonin synthesis and interfere with MT synthesis, which was also metabolized by 5-HT (Li et al. 2005). In the CNS, 5-HT1A receptors among 5-HT receptor subtypes are mainly involved in sleep regulation. Studies have shown that, in addition to expressing dendritic 5-HT1Ars on serotonergic neurons, 5-HT1Ars (pre-synaptic and/or post-synaptic) are particularly concentrated in periventricular hypothalamic and preoptic areas, and the circumventricular organs (Dos Santos et al. 2015). To evaluate the modulation effect of the LD extract on 5-HT receptors, the expression of the 5-HT1A receptors in the hypothalamus of rats was determined. The results showed that LD extract increased the 5-HT1A receptor in the hypothalamus (Figure 6(A)).

GABA, as a main inhibitory neurotransmitter in the mammalian brain, may inhibit arousal systems to promote sleep by binding to the GABA_A_ receptor. There are generally 2 types of GABA receptors: GABA_A_ and GABA_B_. The most important receptor concerning sleep is the GABA_A_ receptor (Gottesmann 2002). It is well-known that activation of GABA_A_ receptors is beneficial for sleep (Abdou et al. 2006). Hence GABA_A_ receptors are key targets in the search for natural resist sleep disorders compounds (Trauner et al. 2008). The results showed that LD extract upregulated the expression of the GABA_A_ receptor (Figure 6(A)). In several studies, it was revealed that exogenous melatonin supplementation increased the GABA content in the hypothalamus, thereby indicating that the sleep regulation effect of melatonin was related to the GABA content in the hypothalamus (Wang et al. 2000), which is also consistent with our findings after LD extract administration. Besides, studies have shown that GABA_A_ receptors (GABA_A_Rs) are of greater relevance to activation of the HPA axis (Brickley and Mody 2012), and a role of GABA in the regulation of the HPA axis in response to stress has been also well established (Decavel and Van den Pol 1990; Cullinan et al. 2008).

Saponins, as characteristic constituents of the genus *Lilium* (Munafo and Gianfagna 2015), have many pharmacological activities, including anti-depressant activities (Liu et al. 2017), anti-inflammation (Zhao et al. 2019), etc. In addition, it has been reported that total saponins in *L. lancifolium* and *L. brownii* can prolong the sleep time of mice caused by pentobarbital sodium (Li et al. 2012, Wang et al. 2015). Saponins may also be the main active ingredient of alleviating insomnia for LD. The total saponin content of LD in this study was 3.11%, which was determined by ultraviolet-visible (UV) spectrophotometry using diosgenin as the control.

## Conclusions

Our findings show that LD extract can regulate the hypothalamic related neurotransmitters, melatonin, and homeostasis of HPA axis, thereby relieving symptoms of insomnia induced by injection PCPA in rats. Accordingly, *L. davidii* can be considered to develop health-care food or novel drugs to cope with the increasing number of insomniacs.
